# MUC1c Regulates Cell Survival in Pancreatic Cancer by Preventing Lysosomal Permeabilization

**DOI:** 10.1371/journal.pone.0043020

**Published:** 2012-08-13

**Authors:** Sulagna Banerjee, Nameeta Mujumdar, Vikas Dudeja, Tiffany Mackenzie, Tara K. Krosch, Veena Sangwan, Selwyn M. Vickers, Ashok K. Saluja

**Affiliations:** 1 Division of Basic and Translational Research, Department of Surgery, University of Minnesota, Minneapolis, Minnesota, United States of America; 2 Department of Pharmacology, University of Minnesota, Minneapolis, Minnesota, United States of America; 3 Masonic Cancer Centre, University of Minnesota, Minneapolis, Minnesota, United States of America; University of Nebraska Medical Center, United States of America

## Abstract

**Background:**

MUC1 is a type I transmembrane glycoprotein aberrantly overexpressed in various cancer cells including pancreatic cancer. The cytosolic end of MUC1 (MUC1-c) is extensively involved in a number of signaling pathways. MUC1-c is reported to inhibit apoptosis in a number of cancer cells, but the mechanism of inhibition is unclear.

**Method:**

Expression of MUC1-c was studied in the pancreatic cancer cell line MIAPaCa-2 at the RNA level by using qRTPCR and at the protein level by Western blotting. MUC1-c expression was inhibited either by siRNA or by a specific peptide inhibitor, GO-201. Effect of MUC1-c inhibition on viability and proliferation and lysosomal permeabilization were studied. Association of MUC1-c with HSP70 was detected by co-immunoprecipitation of MUC1-c and HSP70. Localization of MUC1-c in cellular organelles was monitored by immunofluorescence and with immuno- blotting by MUC1-c antibody after subcellular fractionation.

**Results:**

Inhibition of MUC1-c by an inhibitor (GO-201) or siRNA resulted in reduced viability and reduced proliferation of pancreatic cancer cells. Furthermore, GO-201, the peptide inhibitor of MUC1-c, was effective in reducing tumor burden in pancreatic cancer mouse model. MUC1-c was also found to be associated with HSP70 in the cytosol, although a significant amount of MUC1 was also seen to be present in the lysosomes. Inhibition of MUC1 expression or activity showed an enhanced Cathepsin B activity in the cytosol, indicating lysosomal permeabilization. Therefore this study indicates that MUC1-c interacted with HSP70 in the cytosol of pancreatic cancer cells and localized to the lysosomes in these cells. Further, our results showed that MUC1-c protects pancreatic cancer cells from cell death by stabilizing lysosomes and preventing release of Cathepsin B in the cytosol.

## Introduction

Pancreatic cancer is the fourth leading cause of cancer death in both men and women in the United States. Most pancreatic cancers are ductal adenocarcinoma. The 5-year survival rate for patients with localized disease after surgical resection is 20% and for those with metastatic disease, the survival rate is extremely low. Although significant resources have been committed to improving the survival of patients with pancreatic cancer in the past few decades, there has been no significant improvement in these numbers [1]. The poor survival rate is attributed to the late detection of pancreatic cancer and the extreme resistance of the tumor cells to any chemotherapeutic strategies. For this reason, elucidation of the mechanism of resistance of pancreatic cancer cells is a prime research focus, as it may lead to development of novel therapeutic modalities.

Mucins are transmembrane glycoproteins, present on the surface of various mucosal epithelial and hematopoietic cells, and are reported to be overexpressed in a number of adenocarcinomas [2]. MUC1 is one of the mucins that is associated with poor prognosis, malignant transformation of tumor cells, and resistance to genotoxic anti-cancer agents [3], [4]. MUC1 is also associated with invasion [5]–[7], [8], controlling several cellular signaling pathways [9] and tumor progression [10]. Lack of MUC1 has been correlated with decreased proliferation, invasion, and mitotic rates both *in vivo* and *in vitro* in pancreatic cancer [11].

MUC1 is synthesized as a single peptide that undergoes cleavage into two subunits, subsequently forming a stable non-covalent heterodimer consisting of an extracellular domain and a cytoplasmic tail [12], [13]. The extracellular domain of MUC1 is composed of variable number tandem repeats (VNTR) modified by extensive O-glycans, and acts as a physical barrier against the extracellular milieu. The cytoplasmic tail of MUC1 (MUC1-c) consists of a 58 amino acid extracellular domain, a 28 amino acid transmembrane domain and a 72 amino acid cytoplasmic domain. This cytoplasmic domain, (designated MUC1-c) interacts with β-catenin, the major effector of the canonical Wnt signaling pathway [14], [15], and induces anchorage-independent growth and tumorigenicity [16], [17]. Interaction with β-catenin promotes localization of MUC1 to the nucleus, where MUC1-c interacts with various transcription factors and activates a number of growth and survival pathways [18]–[24], thereby repressing several cell death pathways [25]–[29].

The overexpression of MUC1, as found in human tumors, is associated with localization of MUC1-c to mitochondria [3]. The functional significance of mitochondrial MUC1-c localization is supported by the demonstration that MUC1 attenuates DNA damage-induced release of mitochondrial apoptogenic factors and the apoptotic response [3]. Since MUC1 does not have the classic mitochondrial localization signature, the mitochondrial targeting of MUC1-C is mediated by its interaction with cytosolic chaperones such as HSP70 and HSP90 [30]. As the newly-synthesized and -cleaved MUC1-c is in the cytosol with an exposed hydrophobic transmembrane domain [31], [30], these chaperones are able to bind to it and deliver it to mitochondria [32]. Association of MUC1-c with heat shock proteins after activation with heregulin and its subsequent targeting to the mitochondrial outer membrane has been reported [33]. As a component of the mitochondrial outer membrane, MUC1-c attenuates loss of the transmembrane potential in response to genotoxic stress [34], [3], and confers resistance to death in the cellular response to DNA damage, reactive oxygen species, hypoxia, or activation of the death receptor superfamily [34].

Though the downstream pathways of cell death as a consequence of MUC1 inhibition have been studied in a number of cancers, the mechanism of initiation of mitochondrial depolarization (following MUC1 inhibition) leading to activation of apoptotic pathways is not clear in pancreatic cancer. Mitochondrial membrane depolarization can be the result of a number of events. Damage of the nuclear DNA or endoplasmic reticulum both induce apoptotic cell death that is dependent on mitochondrial depolarization and caspase activation [35]. Similar depolarization and initiation of cell death pathways can also be triggered from the lysosomes. Lysosomal damage in response to genotoxic agents is reported to initiate cell death by release of the hydrolytic enzymes from this organelle into the cytoplasm [36], [37]. Lysosomes contain many different types of hydrolytic enzymes including proteases, lipases, nucleases, glycosidases, phospholipases, phosphatases and sulfatases that usually exert their maximal enzymatic activity at low pH. Lysosomal proteases that have been implicated in cell death are those Cathepsins that remain active at a neutral pH, such as Cathepsin B, Cathepsin D and Cathepsin L. These proteases activate apoptotic effectors such as mitochondria and/or caspases, thereby triggering apoptosis [38], [39].

One of the protein types promoting survival of the pancreatic cells is the family of heat shock proteins, particularly HSP70 [37], [40]–[42]. It has been reported that downregulation of HSP70 results in pancreatic cancer cell death by inducing lysosomal permeabilization, [37] but the exact mechanism of protection has not been studied.

In the current study, we show that HSP70 associates with the C-terminal end of the MUC1 protein (MUC1-c) and transports it to the lysosome, thereby preventing lysosomal permeabilization and promoting cell survival in these tumor cells. We further show that the inhibitor peptide of MUC1 (GO-201) inhibits the activity of MUC1-c and results in reduced cell proliferation and viability of tumor cells both *in vivo* and *in vitro.*

## Results

### Pancreatic Cancer Cells Show Increased Expression of MUC1 Compared with Normal Pancreas

**Figure 1:**
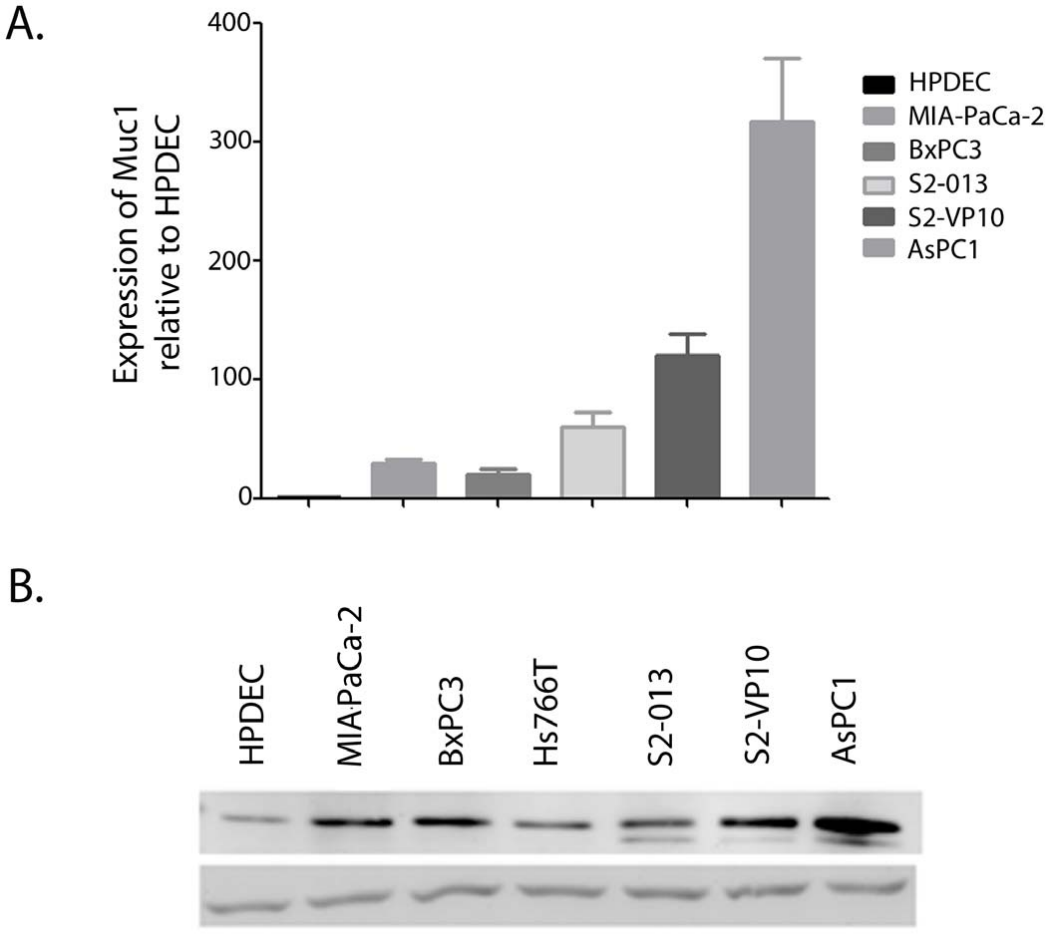
MUC1 is expressed in most pancreatic cancer cell lines and mouse models. mRNA expression levels of MUC1 in several pancreatic cancer cell lines relative to non-tumorigenic HPDEC (A). Data are expressed as mean+/−SEM of 3 independent experiments. *P<.05 (*t* test) as compared with controls. Protein expression levels of MUC1-c in different pancreatic cancer cell line and non-tumorigenic HPDEC (B).

Expression of MUC1 was studied in several pancreatic cancer cell lines at both the RNA and protein level. The expression of MUC1 mRNA was found to be significantly higher in all pancreatic cancer cell lines than in the non-tumorigenic human pancreatic ductal cells (HPDEC) (Fig. 1A). Similar elevated expression of MUC1-c protein was seen in all the tumor cell lines when compared with the non-tumorigenic HPDECs. A correlation was found to exist between aggressiveness of the cell line and expression of MUC1 in pancreatic cancer (Fig. 1B). Higher expression of MUC1 was observed in more invasive and metastatic cell lines S2-013 and S2-VP10 or AsPC1 (derived from ascites) than in cell lines obtained from primary tumors (MIAPaCa-2, BXPC3 and Hs776T).

### Inhibition of MUC1 Leads to Reduced Proliferation and Cell Death

**Figure 2:**
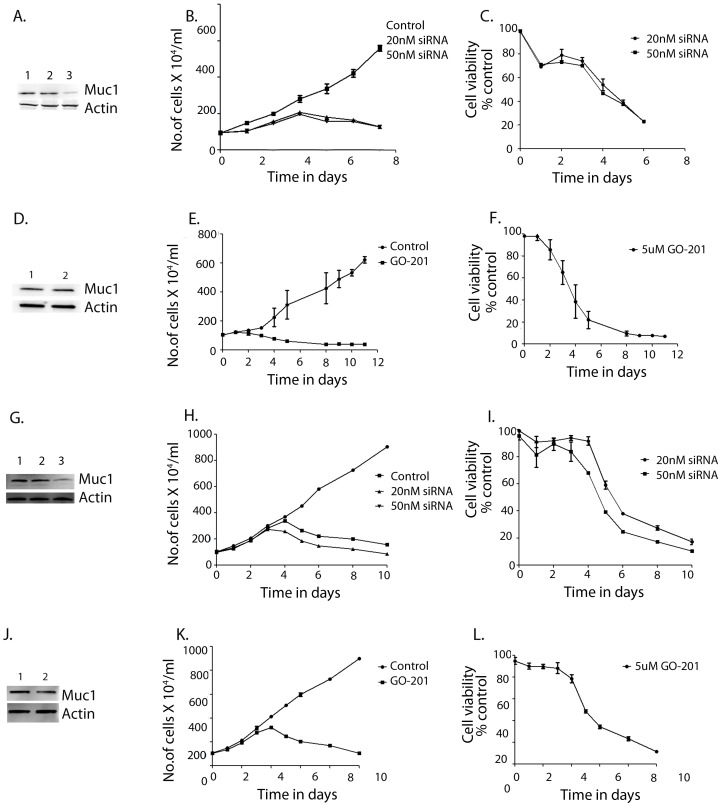
Inhibition of MUC1 expression or activity results in reduced proliferation and cell viability. MUC1 expression was inhibited by siRNA in MIAPaCa-2 (A) Lanes 1–3 show control MIAPaCa-2, MIAPaCa-2 transfected with non-specific siRNA and MUC1 siRNA respectively. Both proliferation (B) and viability (C) were reduced with decreased expression of MUC1 in MIAPaCa-2 cells. On treatment with MUC1-C activity inhibitor GO-201, which inhibits the signaling activity of this protein, no change was seen in expression levels of MUC1 in MIAPaCa-2 cells (D). Lane 1 was untreated MIAPaCa-2 cells and lane 2 was MIAPaCa-2 treated with GO-201. Proliferation (E) and viability (F) were seen to be decreased. Similarly, in another cell line, AsPC-1, transfection with siRNA showed decreased protein levels (G) where Lane 1 is control AsPC-1, Lane 2: non-specific siRNA transfected AsPC-1 and Lane 3 is MUC1 siRNA-transfected AsPC1. Both proliferation (H) and viability (I) were reduced. On treatment with the inhibitor GO-201, there was no change in protein levels (J): Lane 1 was untreated AsPC-1 cells and lane 2 was AsPC-1 treated with GO-201. Proliferation (K) and viability (L) were seen to be reduced. Data are expressed as mean+/−SEM of 3 independent experiments. *P<.05 (*t* test) as compared with controls.

To study if MUC1 was an essential protein controlling cell survival in pancreatic cancer, its expression was inhibited using siRNA against MUC1 in MIAPaCa-2 (Fig. 2A–C) and AsPC1 (Fig. 2G–I) cells. In parallel we also used GO-201, a peptide fragment that inhibits activity of MUC1-c, thereby leading to deregulation of downstream signaling activity and triggering cell death in pancreatic cancer cell lines MIAPaCa-2 (Fig. 2D–F) and AsPC1 (Fig. 2J–L). Both inhibition of MUC1 expression by siRNA (Fig. 2 A, G) as well as inhibition of MUC1-c activity by GO-201 led to reduced proliferation and increased cell death in pancreatic cancer cells. MIAPaCa-2 cells were seen to respond more to inhibition of MUC1 either by siRNA or by GO-201 (Fig. 2A–F) compared to AsPC1 cells (Fig. 2G–L). As expected, GO-201, the peptide inhibitor for MUC1-C, did not alter the expression level of the protein (Fig. 2D, J). Cell death was seen to be caspase-mediated (Figure S1).

### MUC1 Associates with HSP70 in the Cytosol and Localizes in the Lysosomes

**Figure 3:**
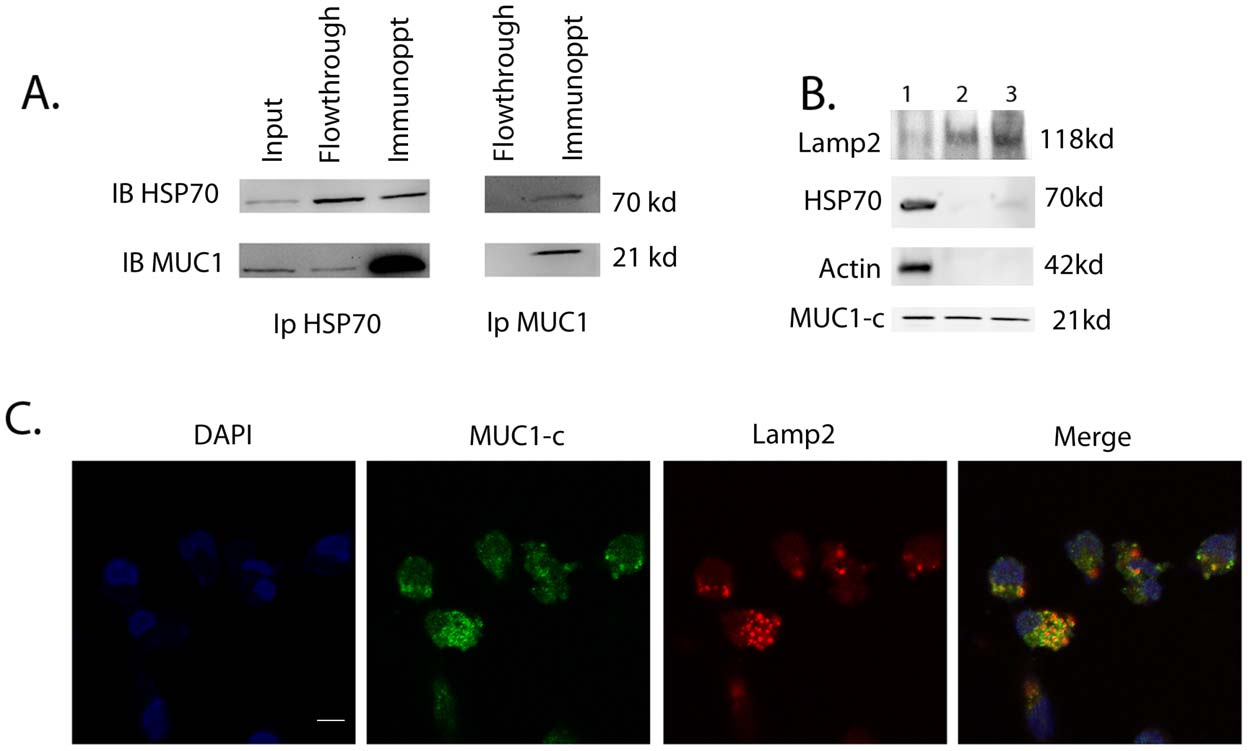
MUC1-c associates with HSP70 and localizes in the lysosomes. Immunoprecipitation with MUC1 and HSP70 showed the two proteins to be associated (A). Lanes 1, 2 and 3 show total protein levels, HSP70 and MUC1 in the flowthrough and the two proteins immunoprecipitated by HSP70 antibody respectively. Lanes 4 and 5 show flowthrough and immunoprecipitates by MUC1 antibody. MUC1-c was found to be localized to the lysosomes in MIAPaCa-2 cells, though almost equal amounts of MUC1-c were also cytosolic (B). Lamp2, a resident lysosomal protein, and actin, a predominantly cytosolic protein, were used as fractionation markers. Lane 1 is the cytosolic fraction. Lane 2 is a crude lysosomal fraction, which is further enriched in Lane 3. HSP70, though associated with MUC1, was seen to be present predominantly in the cytosol. Immunofluorescence confirmed co-localization of MUC1-c and Lamp2 in lysosomes (C).

Heat shock protein 70 (HSP70) is highly overexpressed in pancreatic cancer and its downregulation by inhibitors and siRNA has been seen to induce pancreatic cancer cell death [37]. However, the exact role played by HSP70 in survival of pancreatic cancer cells is unknown. At the same time, MUC1 is known to be overexpressed in pancreatic cancer and play a role in cell survival in a number of cancer cell lines, but the exact mechanism has been elusive [11], [43], [44]. Since HSP70 is a chaperone, it has the ability to bind to and sequester the cytosolic MUC1-c thereby targeting it to different cellular organelles, where MUC1-c could regulate the various anti-apoptotic pathways to protect pancreatic cancer cells from apoptotic cell death. It has been shown previously that MUC1 binds to HSP70 and HSP90 and is targeted to the mitochondrial outer membrane [33]. To see whether MUC1-c is associated with HSP70 in pancreatic cancer cells, we tested for co-precipitation of MUC1 and HSP70 in pancreatic cancer cells. MUC1-c and HSP70 were found to co-immunoprecipitate, onfirming that HSP70 and MUC1 indeed interact in pancreatic cancer cells (Fig. 3A).

MUC1 is extensively present on the cell surface of pancreatic cells. However, the 72 amino acid C-terminal end of the protein undergoes auto-cleavage and localizes to different cellular organelles in response to a differential signaling mechanism. To see if MUC1-c was indeed transported by HSP70 to various cellular compartments, we performed immunofluorescence with organelle specific markers along with subcellular fractionation followed by immunoblotting with anti-MUC1 antibody to look for its localization. The integrity of the different fractions and the purity of the organellar preparations were confirmed by using different markers (Lamp2 for lysosomes and Actin for cytosol). Although HSP70 was seen to co-precipitate with MUC1-c (Fig. 3A), it was seen to be localized in the cytosol and not in the lysosomes, whereas MUC1-c was found to be localized in both cytosol and lysosomes (Fig. 3B). Localization of MUC1 in lysosomes was further confirmed by co-staining pancreatic cancer cells with the lysosomal marker Lamp2 (Fig. 3C). Immunofluorescence showed that MUC1 and Lamp2 co-localize with a Pearson’s Correlation of 0.75 (Fig. 3C). This indicated that MUC1-c associated with HSP70 in the cytosol and localized in the lysosomes. Some MUC1-c was also seen to localize in the mitochondria, thereby confirming the earlier study by Ren et al that MUC1-c is targeted to mitochondria by HSP70 [3] (Figure S2).

### Inhibition of MUC1-c or HSP70 Results in Lysosomal Permeabilization Leading to Cell Death

**Figure 4:**
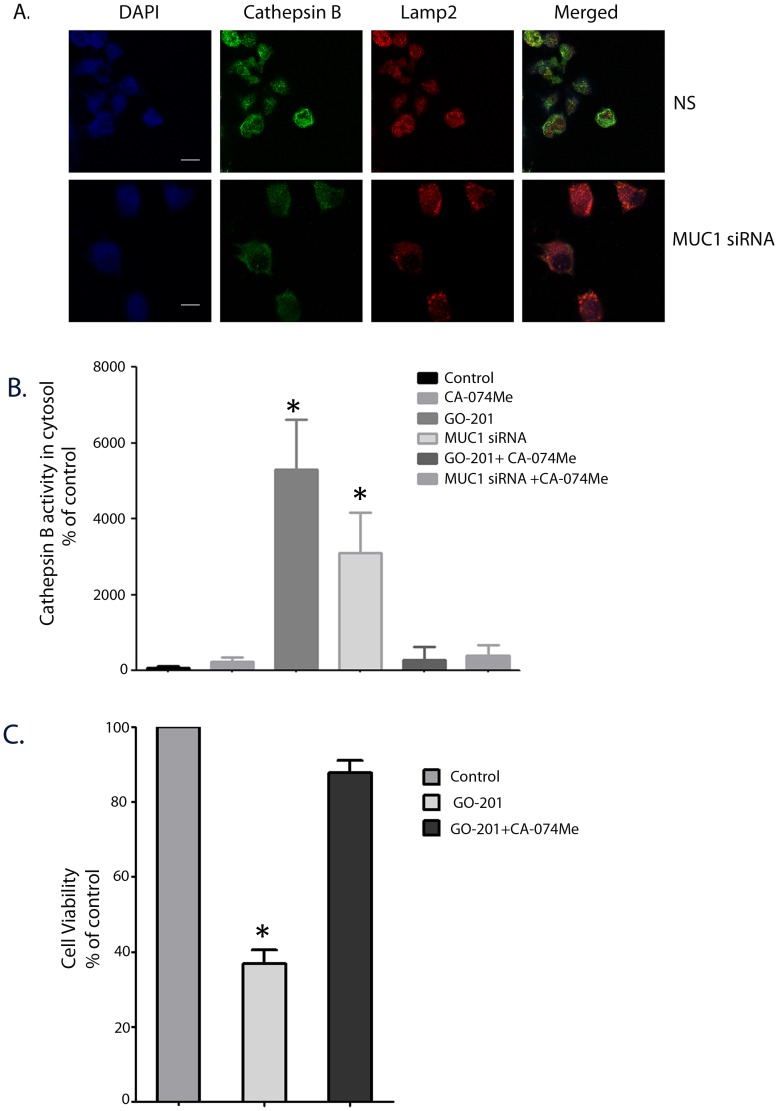
Inhibition of MUC1 leads to lysosomal permeabilization. Inhibition of MUC1 by siRNA resulted in lysosomal permeabilization as manifested by Cathepsin B release in the cytosol (A). Loss of colocalization of Cathepsin B and Lamp2 in MUC1-silenced cells was observed compared with non-silencing control. Biochemical assay for Cathepsin B activity in cytosol showed increased activity in both MUC1 silenced cells and cells treated with GO-201 (B). The cell death process induced by GO-201 was arrested on using a Cathepsin B inhibitor CA-O74Me (C). Data are expressed as mean+/−SEM of 3 independent experiments. *P<.05 (*t* test) as compared with controls.

**Figure 5:**
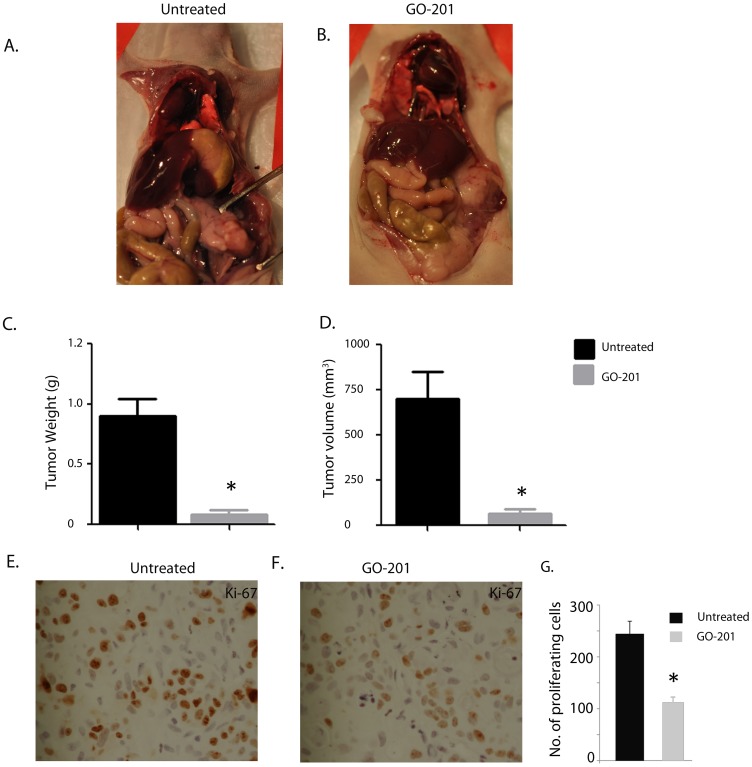
Pharmacological inhibitor of MUC1-CT, GO-201, showed marked reduction of tumor burden in mouse model of pancreatic cancer. Orthotopic mouse model for pancreatic cancer with AsPC-1 cells showed huge pancreatic tumor in untreated mice (A) whereas GO-201 had very little no tumor in their pancreas. Reduction of tumor weight (C) and volume (D) was observed after treatment with GO-201. *represents p<0.05 as compared with controls. Representative tissue sections from mice with pancreatic tumor (orthotopic model with AsPC1 cells) in the control group stained with Ki-67 showed extensive staining indicating actively proliferating tumor cells (E), in comparison to less staining in GO-201 slides showing loss in proliferation (F). The total number of cells stained with Ki-67 was counted in at least 10 fields in both samples and the average number of proliferating cells in both sets were plotted (G) *P<.05 (*t* test) as compared with controls.

Lysosomal permeabilization is often thought to precede cell death in response to a stimulus by releasing a number of hydrolytic enzymes in the cytosol, thereby reducing the pH of the cytosol and causing acidosis [45]. Release of lysosomal enzymes such as Cathepsin B and Cathepsin D in turn induce mitochondrial depolarization resulting in the release of cytochrome C and activation of apoptotic pathways [45]. Since a significant fraction of MUC1 was localized in lysosomes, we looked for an effect on lysosomal permeabilization by measuring loss of Cathepsin B-Lamp2 co-localization in the lysosomes following inhibition of MUC1. We further confirmed this phenomena by measuring the cytosolic activity of Cathepsin B in cells after inhibition of MUC1 by siRNA. Our immunofluorescence studies showed a distinct loss in co-localization of CathepsinB and Lamp2 co-staining in MUC1-silenced cells (Fig. 4A). Our biochemical studies showed that inhibition of expression of MUC1 showed an increase in cytosolic Cathepsin B activity indicating that the lysosomal membrane integrity was compromised (Fig. 4B). An almost equal magnitude of cytosolic Cathepsin B activity was observed when HSP70 was inhibited using siRNA (Figure S3). This was similar to an earlier study [37] in our lab reporting that downregulation of HSP70 in pancreatic cancer resulted in cell death by lysosomal permeabilization. This indicated that inhibition of either HSP70 or MUC1 resulted in lysosomal permeabilization. To determine whether this permeabilization was directly related to cell death, we used a cell-permeable Cathepsin B inhibitor (CA-074-Me) and measured the viability of the cells in the presence of a MUC1 inhibitor (Fig. 5C). While the viability of the cells was reduced to almost 40% of untreated cells in the presence of GO-201, cells treated with CA-074Me along with GO-201 were seen to recover and showed a viability of almost 98% of untreated cells (Fig. 4C). It was observed that cells treated with only GO-201 showed reduced growth and proliferation after 3 days of treatment while the ones which were treated with the Cathepsin B inhibitor showed proliferation and growth similar to those of controls. Similar restoration of viability of MIAPaCa-2 cells was also observed when MUC1 expression was inhibited by siRNA (Figure S4). This showed that the effect of GO-201 on Cathepsin B release could be reversed by the use of a Cathepsin B inhibitor (Fig. 4D). This was also confirmed biochemically, in which MUC1 siRNA and GO201 both showed increased cytosolic Cathepsin B activity but treatment with CA-074Me showed a reversal of this activity. This indicated that MUC1 inhibition indeed led to lysosomal permeabilization, which in turn triggered the onset of cell death pathways in these cells.

### Inhibition of MUC1 Expression in an *in vivo* Mouse Model of Pancreatic Cancer Reduces Tumor Progression

GO-201, a peptide inhibitor reported to inhibit the activity of MUC1-c, has been tested for tumor regression in a number of cancer models such as prostate cancer, breast cancer and CML [46]–[48]. However, its efficacy has not been tested in pancreatic cancer models. We used an orthotopic pancreatic cancer mouse model with the aggressive cell line AsPC1 to test the efficacy of GO-201. Twenty athymic nu/nu mice were given intra-pancreatic injections of 2×10^5^ cells. Treatment with intraperitoneal GO-201 (30 mg/kg body weight) was started on 10 randomized mice, beginning on day 4 after tumor cell injection. The remaining 10 mice were injected with saline. The experiment was terminated on day 30 after injection of cells. The mice were euthanized and the tumor burden in both the groups was evaluated. On day 30, all saline animals had tumors (n = 10) (Fig. 5A) whereas in the treated group of n = 10 mice, only 3 of 10 had tumors (Fig. 5B). The tumor weight in the untreated group ranged from 0.6–1.6 g whereas the treated groups had tumors weighing 0.1 g–0.3 g (Fig. 5C). The average tumor volume of the mice in the saline group was 0.9 cm^3^ whereas those treated with GO-201 had an average tumor volume of 0.1 cm^3^ (Fig. 5D). The saline group also showed extensive metastatic spread of tumors to a number of organs (Table 1). The tissues from the tumors were stained with Ki-67 to compare proliferation rates in the two groups. The untreated group stained extensively with Ki-67, showing active proliferating cells (Fig. 5E). In comparison, the GO-201 treated cells stained very weakly with Ki-67 (Fig. 5F). This suggested that functional inhibition of MUC1-c by GO-201 resulted in significant reduction of tumor burden in mice.

**Table 1:** Evaluation of metastasis in the mouse model for pancreatic cancer.

	Saline (No. of animals showing metastasis)	GO-201 (No. of animals showing metastasis)
Liver	7	0
Lung	6	0
Kidney	2	1
Spleen	5	0

## Discussion

Heat-shock (HSP) 70 proteins function as ATP-dependent chaperones that regulate the folding of newly- synthesized proteins, the assembly of multi-protein complexes and the transport of proteins across cellular membranes [49]. HSP70 is frequently overexpressed in malignant tumors, and this overexpression is associated with a poor therapeutic outcome in a number of human cancers, including breast and pancreatic cancer [50], [37], [40], [41]. HSP70 has a marked cytoprotective effect and inhibits apoptotic signaling by inhibiting mitochondrial permeabilization, by reducing caspase activation or by neutralizing apoptosis inducing factors [51]. HSP70 also localizes to lysosomal membranes [52], [53] and can protect lysosomal membranes against LMP induced by different stimuli such as etoposide, TNF-α and oxidative stress [54]. We have previously shown that HSP70 is overexpressed in pancreatic cancer cells and that its downregulation by either siRNA *in vitro* or by use of an inhibitor *in vivo* induces caspase activation and cell death by apoptosis [37], [42], [55]. This suggests that HSP70 is important for the survival of pancreatic cancer cells. Many probable mechanisms have been proposed for the pro-survival function of HSP70. *In vitro* studies have suggested that HSP70 may interfere directly with the apoptosis-signaling machinery downstream of mitochondria by preventing apoptosome formation and caspase activation [56]. Recent studies using cellular death models have pointed out that HSP70-mediated inhibition of caspase-dependent apoptosis occurs upstream of mitochondrial outer membrane permeabilization (MOMP) and cytochrome c release [57], [58]. Previous studies from our group also suggest that the inhibition of HSP70 expression by different inhibitors or HSP70 siRNA leads to caspase activation and apoptosis, suggesting that HSP70 inhibits caspase-dependent apoptosis in pancreatic cancer cells by influencing cytosolic calcium reserves and causing lysosomal permeabilization. However, the molecular mechanism of this phenomenon has remained elusive [37].

MUC1-c has been shown to be an oncoprotein responsible for transformation and tumorigenicity in a number of cancer cells. MUC1-c also plays an intensive role in cellular signaling following a number of phosphorylation events. Several reports also show an accumulation of MUC1-c in the cytosol of cancer cells. It has been shown previously that the MUC1-c associates with heat shock proteins and is targeted to the mitochondrial outer membrane [33], [59]. Thus a close association of HSP70 and MUC1 exists in tumor cells.

In an attempt to understand the molecular mechanism of cell survival and the role of HSP70 and MUC1 in pancreatic cancer cells, we looked into the association of HSP70 and MUC1. Since the predominant function of HSP70 in any cell is its chaperone activity, we hypothesized that HSP70 acts as a carrier of MUC1-c to different cellular compartments such as lysosomes and mitochondria, resulting in protection of these compartments and preventing cell death. Our current study showed that HSP70 was associated with MUC1 and that it co-precipitated with MUC1-c (Fig. 3A). It also showed that though MUC1-C was predominantly localized in the cytoplasm, a significant amount was also present in the lysosomes and mitochondria (Fig. 3 B, C; Figure S2). Inhibition of MUC1-c expression by siRNA or an inhibition of activity by using the peptide inhibitor GO-201 resulted in decreased proliferation and viability of pancreatic cancer cells (Fig. 2) and induced caspase activity in these cells, indicating an activation of apoptotic pathways (Figure S1). This inhibition also caused lysosomal permeabilization of pancreatic cancer cells leading to Cathepsin B release and a high cytosolic Cathepsin B activity (Fig. 4B, C). Cathepsin B expression in cells transfected with non-specific siRNA show distinct staining in lysosomal compartments (Figure 4 A). This distinct staining pattern is seen to be diffused once cells are transfected with Muc1 siRNA. This is owing to the release of Cathepsin B protein in the cytosolic compartment as assayed biochemically(Figure 4B). Similar lysosomal permeabilization was also observed when HSP70 expression was inhibited by siRNA (Figure S3). The effect of inhibition of MUC1 expression or activity was seen to be reversed when the cells were co-incubated with the Cathepsin B inhibitor CA-074Me. This indicated that release of Cathepsin B from the lysosomes of pancreatic cancer cells resulted in triggering the cell death pathways. Previous reports from our group show that inhibition of HSP70 by an inhibitor, either triptolide or quercetin, also resulted in similar lysosomal permeabilization and apoptotic cell death [37]. In the same study it was also seen that a Cathepsin B inhibitor was able to reverse the effects of HSP70 inhibition on the pancreatic cancer cells, indicating that lysosomal permeabilization is one of the earlier steps of apoptotic death in these cells.

Classically, lysosomal membrane permeabilization (LMP) has been thought to be involved in cell death mechanisms, though the exact sequential events from LMP to cell death have not been mapped out. Depending on the lethal stimulus, the extent of LMP, the amount and type of Cathepsins released into the cytoplasm, as well as the abundance of Cathepsin inhibitors, LMP can trigger a variety of death-associated morphologies ranging from classical apoptosis to necrosis. Thus, LMP associated with Cathepsin translocation may directly activate calpains and caspases, but LMP may also trigger the classical MOMP–caspase pathway, as well as MOMP- and caspase-independent apoptosis. Which lethal pathways are stimulated by LMP depends on the cell type, the immortalization status and the genetic background of the cells.

In the current study, we propose a model of cell survival in which HSP70 acts as a carrier of MUC1-c by associating with it physically and transporting it to the lysosomes (and mitochondria) in pancreatic cancer cells. The presence of MUC1-c in the lysosomes prevents LMP and keeps the lysosomes intact. However, when HSP70 or MUC1 is inhibited either by siRNA or by a peptide inhibitor of MUC1 (which prevents activity of MUC1 and thus its association with any other molecule), the protection of lysosomes is lost, and there is extensive LMP, leading to a release of Cathepsin B and triggering of cell death. Whether induction of LMP and release of Cathepsin B activates additional apoptotic pathways involving the mitochondria needs to be investigated further.

From a clinical perspective, a more specific approach in exploiting MUC1-c-mediated cell survival is to develop therapeutic agents that interact directly with MUC1-c and block its function. MUC1-c forms oligomers through the CQC amino acid motif in its cytoplasmic domain, and activity is necessary for MUC1-C nuclear transport [60], [61]. The peptide inhibitor GO-201, which contains the CQC motif, has been designed and tested in a number of cancer cells to block oligomerization of MUC1 and thus inhibit its signaling activity and function [18], [46]–[48]. In the current study, we used GO-201 on an orthotopic pancreatic cancer mouse model to test its efficacy on the pancreatic tumor. The aggressive pancreatic cancer cell line AsPC1 was used to generate orthotopic pancreatic tumors. GO-201 (30 mg/kg) was injected intraperitoneally everyday for 26 days and the experimented was terminated at day 30. The inhibition of MUC1 function by GO-201 was seen to prevent tumor growth and progression in the treatment group significantly as compared with the untreated group. In previous studies GO-201 has been shown to be a specific MUC1 inhibitor that has no effect on any protein other than MUC1 [47]. Our earlier studies had shown that by using inhibitors of HSP70, pancreatic tumors could be regressed *in vivo* [60]. In continuation of those investigations, this study also showed that inhibition of MUC1 *in vivo* could decrease tumor burden in mice, thereby confirming that the MUC1-HSP70 association is an essential factor in the survival of pancreatic cancer cells. Further, inhibition of this association by inhibition of either HSP70 expression or MUC1 activity could be of significant therapeutic value in pancreatic cancer.

## Materials and Methods

### Ethics Statement

All procedures were approved by the University of Minnesota Institutional Animal Care and Use Committee (IACUC).

### Cell Culture

Pancreatic cancer cells from the MIAPaCa-2 (ATCC, Manassus, VA) and BxPC3 (ATCC, Manassus, VA) lines were grown and propagated in Dulbecco’s modified Eagle’s medium (Life Technologies, Carlsbad, CA) supplemented with 10% FBS (Life Technologies, Carlsbad, CA) and 100 units/ml penicillin and 100 µg/ml streptomycin (Life Technologies, Carlsbad, CA);Hs766T (ATCC, Manassus, VA) cell lines were grown as sub-confluent monolayer cultures in Dulbecco’s modified Eagle’s medium/Ham’s F-12 medium (1∶1) (Life Technologies, Carlsbad, CA) supplemented with 10% Fetal Calf Serum (Life Technologies, USA), 100 units/ml penicillin and 100 µg/ml streptomycin; S2013 and S2VP10 cell lines (a gift from Prof. Masato Yamamoto’s lab) [62] were cultured in RPMI medium (Life Technologies, Carlsbad, CA) supplemented with 10% Fetal Bovine Serum and 100 units/ml penicillin and 100 µg/ml streptomycin, while AsPC1 (a gift from Prof. Masato Yamamoto’s lab ) [63] cell lines were cultured in RPMI medium supplemented with 20% Fetal Bovine Serum. Human Pancreatic Ductal epithelial cells (HPDEC) (a gift from Prof. Craig Lodgston, MD Anderson, Texas, USA) [64] were cultured in Keratinocyte Media (Life Technologies, Carlsbad, CA) supplemented with Bovine Pituitary Hormone (Life Technologies, Carlsbad, CA) and EGF (Life Technologies, Carlsbad, CA).

### Quantitative Real Time PCR

Quantitative RTPCR for MUC1 was carried out using primers procured from Qiagen, Valencia, CA (MUC1 Quantitect Primer Assay, Qiagen, Valencia, CA, USA). RNA was isolated from the different cell lines and from the tumor samples from different pancreatic cancer mouse models according to manufacturer’s instruction using Trizol (Life Technologies, Carlsbad, CA). Total RNA (1 ug) was used to perform real-time PCR using the Quantitect Sybr green PCR kit (Qiagen, Valencia, CA, USA) according to the manufacturer’s instructions using an Applied Biosystems 7300 real-time PCR system. All data were normalized to the housekeeping gene 18S (18S Quantitect Primer Assay; Qiagen, Valencia, CA, USA).

### Inhibition of MUC1 by siRNA and Peptide Inhibitor GO-201

ON-TARGET plus SMART Pool human HSP70 siRNA (Dharmacon Inc, Lafayette, CO) and human MUC1 siRNA (Qiagen, Valencia, CA) were used to silence expression of HSP70 and MUC1 in the pancreatic cancer cell lines MIAPaCa-2, S2013, AsPC1. Transfections were done at 60% confluency of cells, using Hiperfect (Qiagen, Valencia, CA) according to manufacturer’s instruction.

MUC1 oligomerization and activity was inhibited using the peptide inhibitor GO-201 [NH_2_-RRRRRRRRRCQCRRKNYGQLDIFP] (Sigma, St. Louis, MI) at a concentration of 5 uM every day for 5–8 days. Following treatment cells were harvested and processed further for different assays.

For Cathepsin B inhibition, cells were pre-treated with CA-074Me (Peptides International (Louisville, KY). 10 uM in cell growth medium for 10 min followed by appropriate treatment with GO-201 or MUC1 siRNA.

### Cell Viability and Proliferation Assay

Trypan blue exclusion assays for cell viability and proliferation were performed every day following siRNA or GO-201 treatment. Three different readings were taken and the average values were plotted to estimate the proliferation rate of the cells after MUC1 inhibition. Viability was calculated by using both CCK8 reagent (Dojindo Molecular Technologies (Gaithersburg, MD) and trypan blue (Life Technologies, Carlsbad, CA) exclusion and expressed after normalizing to untreated cells. Caspase assay was performed using the Caspase Glo 3/7 kit (Promega, WI) according to manufacturer’s protocol.

### Immunoprecipitation, Western Blotting and Hybridization

Cell lysates were prepared by resuspending cells in RIPA buffer (Boston Bioproducts, Boston, MA) supplemented with protease inhibitors for 30 min at 4°C and cleared by centrifugation for 30 min at 13,000 g. Supernatants were collected and stored at −80°C. For immunoprecipitation, Anti-MUC-1(Catalog Number SAB2101529 Sigma, St.Louis, MI) and anti-HSP70 (Assay Design, Ann Arbor, MI) antibodies were conjugated to NHS-activated resin in Direct IP kit (Thermo Scientific, Rockford, IL) and 1 mg of total lysate was loaded on conjugated antibody. Immunoprecipitation and elution was performed according to the manufacturer’s instructions. Equal volumes of input, flowthrough and immunoprecipitated proteins were separated on SDS-PAGE and transferred. HSP70 and MUC1 immunoprecipitated samples were both hybridized with anti-HSP70 (Assay Design, Ann Arbor, MI) and anti-MUC1 (Sigma, St.Louis, MI) antibody to assess the co-immunoprecipitation.

For regular Western blotting for checking protein levels, total protein concentration was determined using the BCA Assay kit (Thermo Scientific, Rockford, IL). Anti MUC1-c antibody (Sigma, St.Louis, MI) raised in rabbit was used for detecting MUC1 levels in the cell lysates.

### Isolation of Different Cellular Compartments

For analyzing the sub-cellular localization of MUC1-c in treated and untreated cells, they were fractionated for lysosomes, mitochondria and nucleus. Lysosomes were isolated using the lysosome isolation kit (Sigma, St.Louis, MI) using differential centrifugation according to the manufacturer’s protocol. Fractionation and purity of lysosomes was assessed using the lysosomal marker Lamp2 (Santa Cruz Biotechnology, Santa Cruz, CA), and the cytosolic marker B-actin (Santa Cruz Biotechnology, Santa Cruz, CA). All fractions were separated using SDS-PAGE, transferred to nitrocellulose membrane and hybridized with the different antibodies to test for subcellular localization.

### Confocal Microscopy

Pancreatic cancer cells were grown in chamber slides and treated with non-specific siRNA or MUC1 siRNA for 24 hours, fixed with 2% paraformaldehyde for 30 minutes, and permeabilized with 0.2% saponin for 5 minutes. The cells were incubated with a 1∶200 dilution of mouse monoclonal anti– Cathepsin B antibody (Santa Cruz Biotechnology, Santa Cruz, CA) and 1∶300 dilution of rabbit polyclonal anti-Lamp2) antibody (Santa Cruz Biotechnology, Santa Cruz, CA) for 2 hours at 4°C. For localization studies, the pancreatic cancer cells were grown in chamber slides for 48 h, fixed with 4% paraformaldehyde for 15 min at room temperature and permeabilized with 0.1% Triton X100. Anti-MUC1-c antibody (Sigma, St.Louis, MI) was used at a dilution of 1/200 for 1 h at room temperature. Cytochrome C (BD Pharmingen, San Diego, CA) was used as a mitochondrial marker, and Lamp2 (Santa Cruz Biotechnology, Santa Cruz, CA) was used as a lysosome marker. After three 5-minute washes with phosphate-buffered saline containing 0.02% Tween-20, cells were incubated with secondary antibodies: 1/1200 dilution of Alexa-488-conjugated donkey anti-mouse IgG (Molecular Probes, Carlsbad, CA) and 1/1500 dilution of Alexa-555-conjugated donkey anti-rabbit IgG (Molecular Probes, Carlsbad, CA) for 50 minutes at 4°C. The slides were washed and mounted using Prolong Gold anti-fade agent (Molecular Probes, Carlsbad, CA). Immunofluorescence images were obtained on a Nikon Eclipse Ti confocal microscope (Nikon, Melville, NY) using a 40X oil immersion objective.

### Cathepsin B Activity Assay

To measure cytosolic Cathepsin B activity, the cytosolic fraction was extracted by incubating cells with an extraction buffer (250 mM sucrose, 20 mM HEPES,10 mM KCl, 1.5 mM MgCl_2_, 1 mM, EDTA, pH 7.5) containing 20 ug/mL Streptolysin O (Sigma, St. Louis, MI) for 10 minutes on ice. The Streptolysin O concentration and treatment times were optimized to result in complete lysis of the cells without disrupting the lysosomes. Cathepsin B activity was determined using N-carbobenzoxy-arginyl-arginine-naphthylamide as the substrate, according to the method of McDonald and Ellis [65]. Activity was expressed as units/mg protein in each sample.

### *In vivo* Pancreatic Cancer Mouse Model

Female nude mice (4–6 weeks old from Charles River Laboratories) were used for the pancreatic cancer mouse model. All procedures were carried out according to the guidelines of the University of Minnesota Institutional Animal Care and Use Committee (IACUC). Briefly, 2×10^5^ AsPC cells/10 ul Matrigel (BD Biosciences, Chicago, IL) were injected into pancreas of 20 nude mice. After 4 days, treatment with GO-201 was initiated in 10 randomized mice with 30 mg/kg body weight intraperitoneally, while the remaining 10 mice were injected with saline (vehicle). The treated and untreated mice were sacrificed 30 days after introduction of AsPC1 cells. The tumor burden was evaluated by documenting the tumor volume (length × width × depth) and tumor weight following necropsy.

### Immunohistochemistry

For immunohistochemistry, paraffin tissue sections were received mounted on charged slides. The slides were deparaffinized in xylene and hydrated through graded ethanols. Slides were steamed with a Reveal Decloaker (Biocare Medical, Concord, CA) to minimize background staining. Sniper Universal Blocking Sera (Biocare Medical) were used throughout the protocol. The slides were stained using an antibody against Ki-67 (rabbit polyclonal, Thermo Scientific, Rockford, IL). A diaminobenzidine peroxidase substrate kit (Vector Laboratories) was used to reveal staining for Ki-67. The tissue sections were counterstained with Gill’s hematoxylin (Vector Laboratories). The primary antibody was omitted for the negative controls.

### Statistical Analysis

Values are expressed as the mean ± SEM. All *in vitro* experiments were performed at least three times. The significance of the difference between any two samples was analyzed by unpaired Student’s t-test; values of p<0.05 were considered statistically significant.

## Supporting Information

Figure S1 **MUC1 silencing resulted in apoptotic cell death in MIAPaCa-2 and AsPC1 cells.** Increased caspase 3 activity was seen in MIAPaCa-2 and AsPC1 cells after MUC1 silencing. Data are expressed as mean+/−SEM of 3 independent experiments. *P<.05 (*t* test) as compared with controls (non-silencing siRNA).
(TIF)

Figure S2 **MUC1 localized in mitochondria in pancreatic cancer cells.** Immunofluorescence showed MUC1 and cytochrome C to co-localize (A). Some MUC1 was present in the isolated mitochondrial fraction along with cytochrome C (B).
(TIF)

Figure S3 **Silencing HSP70 also results in Cathepsin B release in cytosol.** When HSP70 expression was inhibited by siRNA, an equal amount of Cathepsin B release was observed in the cytosol. Data are expressed as mean+/−SEM of 3 independent experiments. *P<.05 (*t* test) as compared with controls.
(TIF)

Figure S4 **MUC1 silencing resulting in Cathepsin B release leading to cell death is rescued by treatment with CA0674-Me, a Cathepsin B inhibitor.** Reduced viability of MIAPaCa-2 cells was observed after MUC1 silencing. This was reversed on treatment with the Cathepsin B inhibitor CA074-Me. Data are expressed as mean+/−SEM of 3 independent experiments. *P<.05 (*t* test) as compared with controls.
(TIF)
