# Supplementary figures and images for: MUC1c Regulates Cell Survival in Pancreatic Cancer by Preventing Lysosomal Permeabilization
Source: PLoS One. 2012 Aug 13;7(8):e43020. doi: 10.1371/journal.pone.0043020 (PMC3418232; doi:10.1371/journal.pone.0043020)

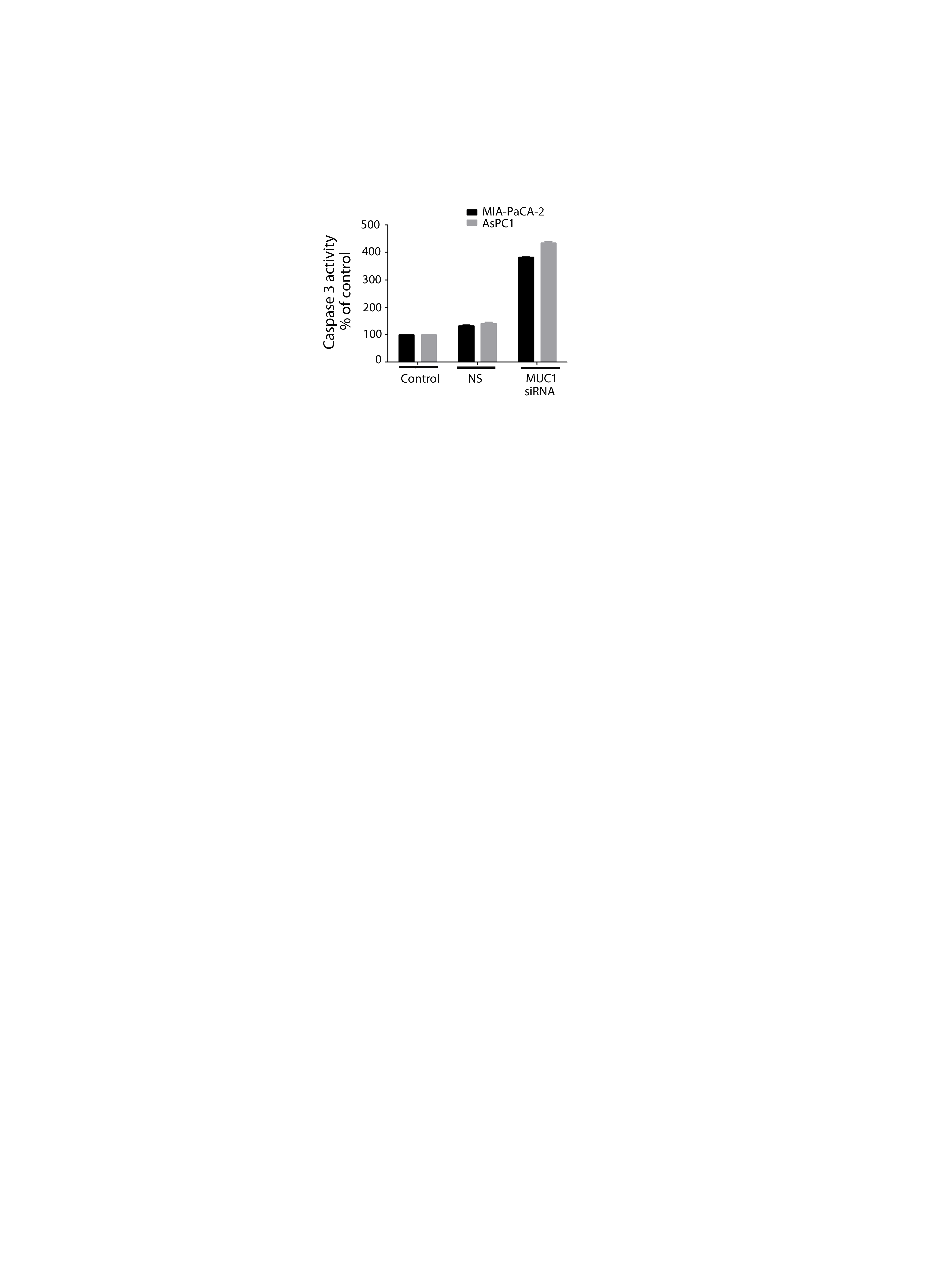

Supplement: Figure S1 — MUC1 silencing resulted in apoptotic cell death in MIAPaCa-2 and AsPC1 cells. Increased caspase 3 activity was seen in MIAPaCa-2 and AsPC1 cells after MUC1 silencing. Data are expressed as mean+/−SEM of 3 independent experiments. *P<.05 (t test) as compared with controls (non-silencing siRNA). (TIF) [file pone.0043020.s001.tif]

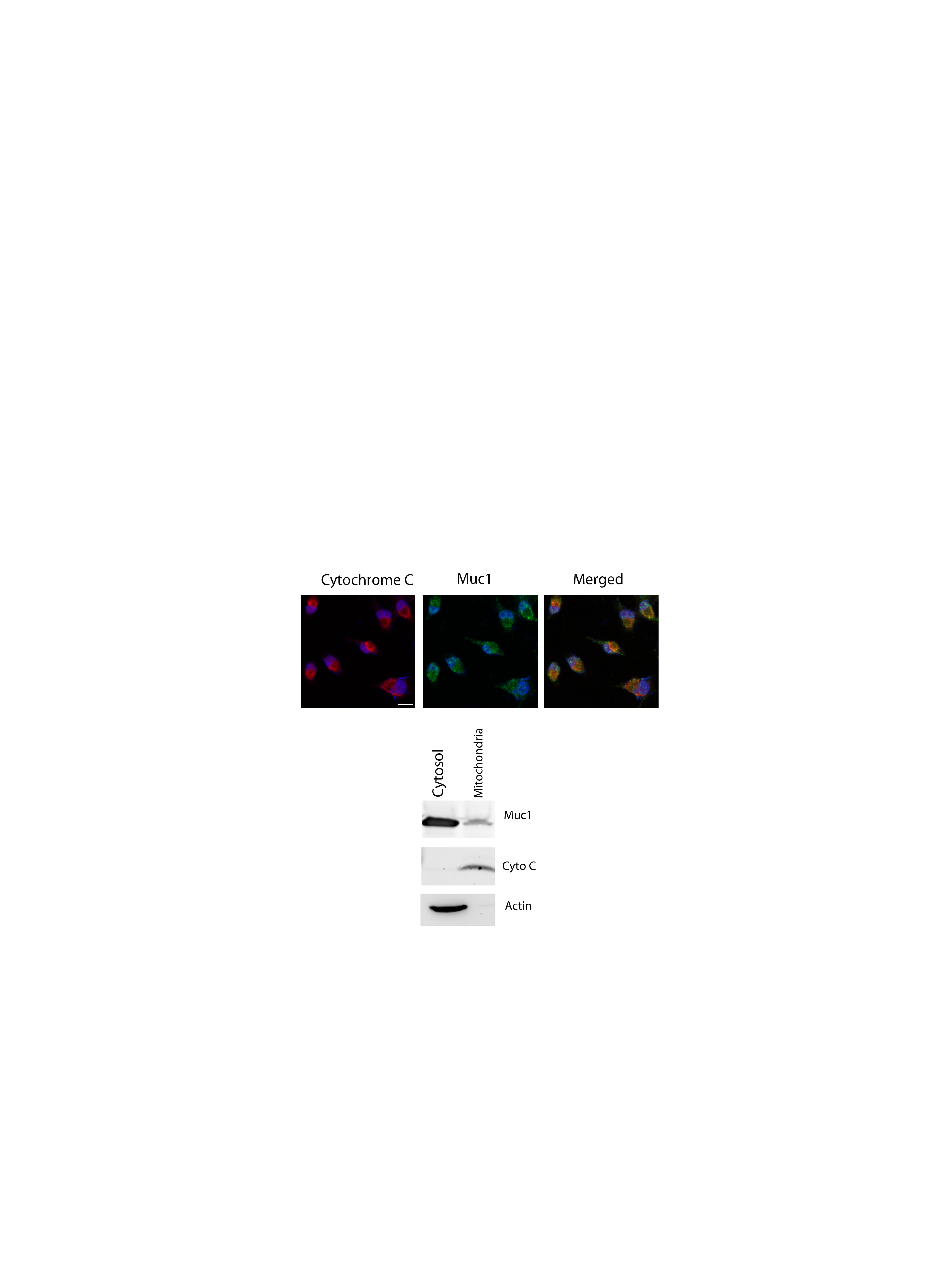

Supplement: Figure S2 — MUC1 localized in mitochondria in pancreatic cancer cells. Immunofluorescence showed MUC1 and cytochrome C to co-localize (A). Some MUC1 was present in the isolated mitochondrial fraction along with cytochrome C (B). (TIF) [file pone.0043020.s002.tif]

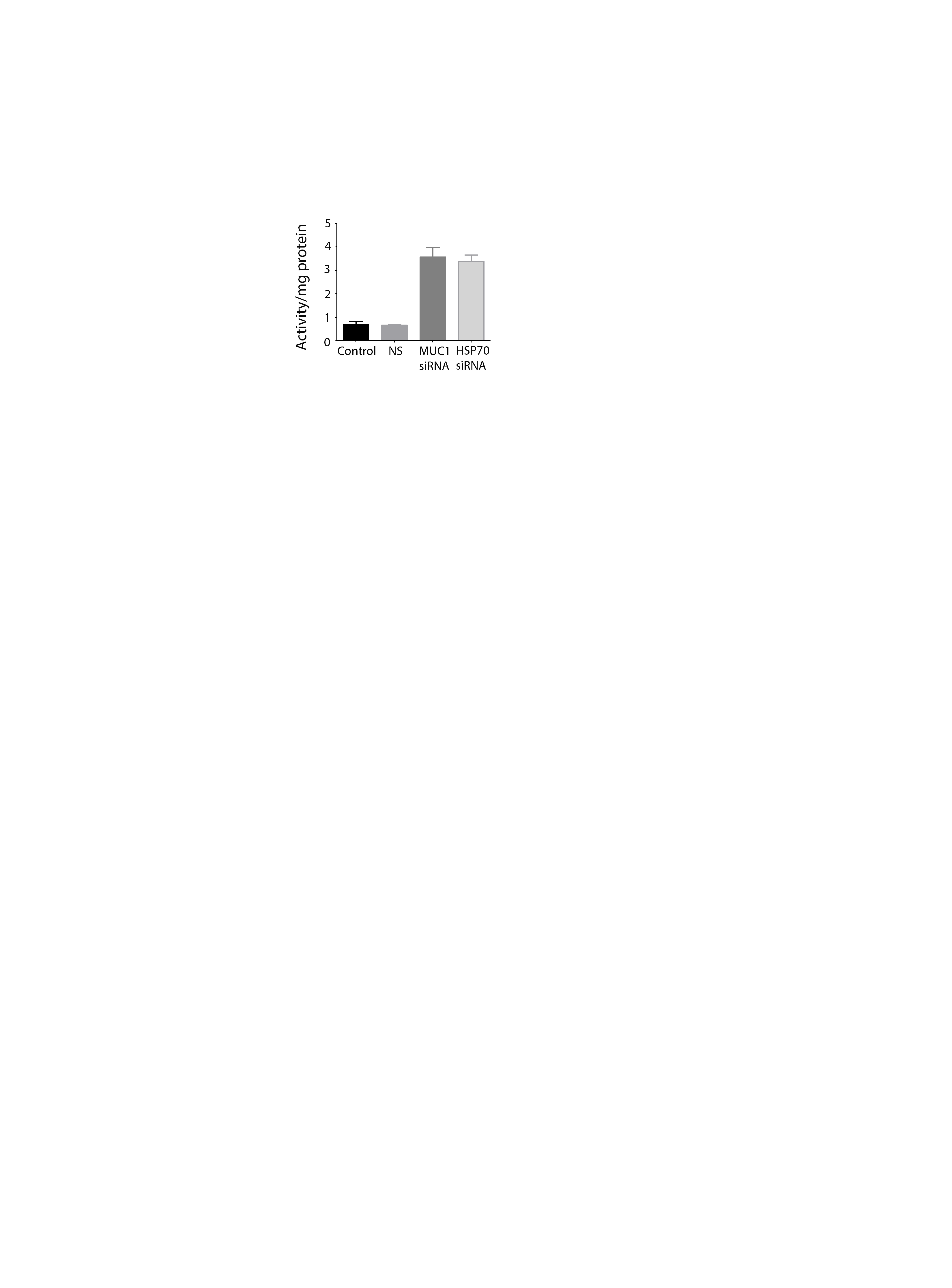

Supplement: Figure S3 — Silencing HSP70 also results in Cathepsin B release in cytosol. When HSP70 expression was inhibited by siRNA, an equal amount of Cathepsin B release was observed in the cytosol. Data are expressed as mean+/−SEM of 3 independent experiments. *P<.05 (t test) as compared with controls. (TIF) [file pone.0043020.s003.tif]

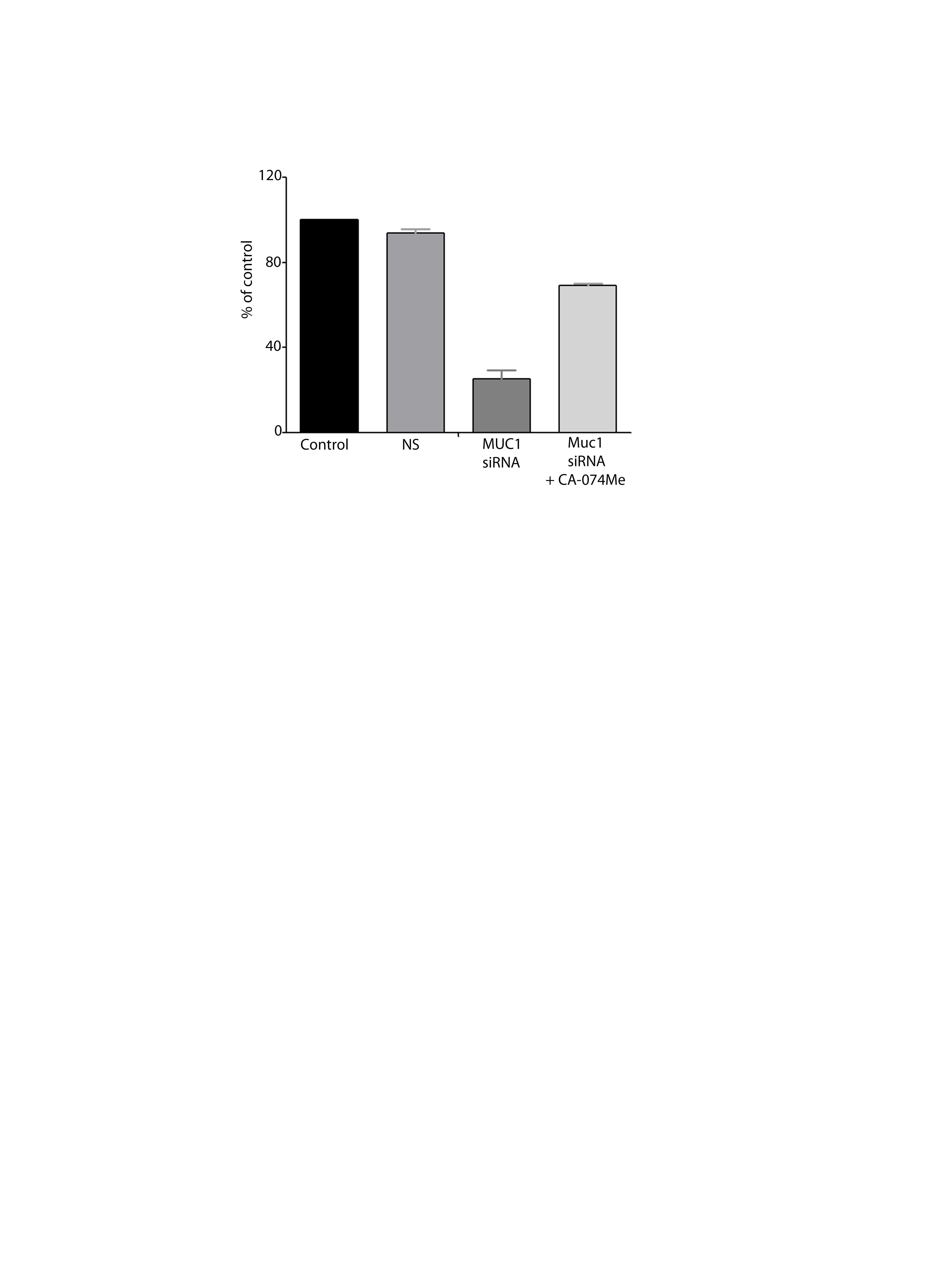

Supplement: Figure S4 — MUC1 silencing resulting in Cathepsin B release leading to cell death is rescued by treatment with CA0674-Me, a Cathepsin B inhibitor. Reduced viability of MIAPaCa-2 cells was observed after MUC1 silencing. This was reversed on treatment with the Cathepsin B inhibitor CA074-Me. Data are expressed as mean+/−SEM of 3 independent experiments. *P<.05 (t test) as compared with controls. (TIF) [file pone.0043020.s004.tif]
